# Medical Education

**Published:** 1904-05-21

**Authors:** 


### The Hospital.

A Journal of

The Medical Sciences and Hospital Administration.

Vol. XXXVI.—No. 921. MAY 21, 1904.

### Medical Education.

Sir Isambard Owen, in delivering the annual Oration before the Medical Society of London on Monday evening, availed himself of the occasion to dwell forcibly upon the difficulties under which medical education is at present conducted in this country, and upon the immense importance to the community of the result which is thus, in some degree, attained less effectively than would be desirable. He pointed out that, in the progress of events, there has gradually come to be an entire separation between the preliminary and the practical parts of the medical curriculum; and that the student of to-day is not encouraged, or even permitted, to enter the wards of a hospital, or to commence the observation of disease, until he has attained a certain degree of proficiency in studies of an introductory character, such as anatomy, physiology, chemistry, and allied branches of knowledge. The effect is that the latter studies may now be pursued at centres of education remote from or unconnected with hospitals, while the progress even recently made in them has been of such a kind as to add very considerably to the cost of the necessary teaching, whether this cost may arise from the character and number of the appliances required or from the necessity of providing suitable remuneration for professors of a very high class. In consequence of these changes, the individual medical schools of London are sorely handicapped in their endeavours to supply effective preliminary instruction, and are rapidly being surpassed in relation to it by the schools of the great provincial cities, some of which are assisted by endowments, while most of them are able to bring together larger, and hence more remunerative, classes than are furnished by the first and second year's men of any single London hospital. The general result is that the preliminary scientific education of the provincial student is often better than that of the London student; while, on the other hand, the advantages of London as a centre of distinctively professional education are great and conspicuous. Sir Isambard Owen paid a just and well-merited tribute to the excellence of many provincial hospitals, and to the skill of many of the physicians and surgeons who practise and teach in them; but, at the same time, he did not hesitate to point out the advantages which the metropolis may justly claim. The attainment of high distinction in a provincial city, as in the case of Syme, or of Lord Lister, or of Sir William Roberts, or of many others who might be named, has not uncommonly been the occasion of removal to London; while the amount of clinical material, the number and variety of the patients and of the diseases to be witnessed in the great city, as well as the diversified forms and methods of competing skill and ingenuity in dealing with them, are of necessity unapproached elsewhere. It would be nothing less than a national misfortune, tending inevitably towards a lowered standard of practical medical education, if the Metropolis were to lose its pre-eminent position as a centre of medical and surgical study and teaching; and the main object of Sir Isambard Owen's discourse was to call attention to this danger, and to urge the claims of the new University of London to the support which will enable it to supply preliminary scientific education of a quality unsurpassed elsewhere, and thus to liberate the hospital schools from the business of providing it and to place them at liberty to devote their whole energies to the work of more advanced teaching. If this could be done the advantages of London as a scientific school would be equal to her advantages as a clinical school, and she would once more assume her right place as the chief centre of medical education in the Empire.

The University of London at present is little more than a name—*vox, et prœterea nihil*—and cannot assume practical form and substance unless or until it is supported by adequate endowments. In continental countries the necessary funds would be given ungrudgingly by governments; in the United States of America they would be furnished by private munificence almost as soon as the necessity for them became declared. In England alone, to the discredit alike of our rulers and of our more wealthy citizens, the urgent want is left unheeded and unsupplied. People in England, as a matter of fact, are not sufficiently educated themselves, in any proper sense of the word, either to appreciate education in others or to contribute cheerfully to its support. Oxford and Cambridge, as Sir Isambard told his audience, no longer hold even a very high place among the world's universities; and "exploded errors in extinct tongues" are still regarded as constituting the crown and finish of intellectual cultivation. Many of those who talk most about education, and some of those whose duty it has now become to supervise and control it, are in reality not only lukewarm but even hostile ; and have as their leading principle a desire to train up the rising generation in the grooves and ruts of an earlier period, and to discourage or prevent any exercise of the reason which might render them scornful of the falsehoods and the claptrap of demagogues, or of the prostitution of religious professions to the purposes of political faction. We are in the dilemma that an extension of true education is an essential preliminary to any adequate realisation of its value; while, at the same time, the neglect of it is a standing menace to the strength and the prosperity of the Empire. Sir Isambard Owen, in calling the attention of the Fellows of the Medical Society to the question, was addressing himself to hearers who would need no conversion to his views, but who would seldom, unfortunately, be in a position to render the aid which the emergency demands. We can only hope that his words will pass beyond the narrow limits of his audience, and that some proportion of them may ultimately alight upon "good ground."

